# Bayesian inference of antigenic and non-antigenic variables from haemagglutination inhibition assays for influenza surveillance

**DOI:** 10.1098/rsos.180113

**Published:** 2018-07-25

**Authors:** Emmanuel S. Adabor, Wilfred Ndifon

**Affiliations:** 1 Research Centre, African Institute for Mathematical Sciences, Cape Town, South Africa; 2 Department of Mathematical Sciences, Stellenbosch University, Stellenbosch, South Africa; 3 Research Department, African Institute for Mathematical Sciences, Next Einstein Initiative, Kigali, Rwanda

**Keywords:** influenza virus, influenza surveillance, haemagglutination inhibitions assays, Bayesian inference

## Abstract

Haemagglutination inhibition (HI) assays are typically used for comparing and characterizing influenza viruses. Data obtained from the assays (titres) are used quantitatively to determine antigenic differences between influenza strains. However, the use of these titres has been criticized as they sometimes fail to capture accurate antigenic differences between strains. Our previous analytical work revealed how antigenic and non-antigenic variables contribute to the titres. Building on this previous work, we have developed a Bayesian method for decoupling antigenic and non-antigenic contributions to the titres in this paper. We apply this method to a compendium of HI titres of influenza A (H3N2) viruses curated from 1968 to 2016. Remarkably, the results of this fit indicate that the non-antigenic variable, which is inversely correlated with viral avidity for the red blood cells used in HI assays, oscillates during the course of influenza virus evolution, with a period that corresponds roughly to the timescale on which antigenic variants replace each other. Together, the results suggest that the new Bayesian method is applicable to the analysis of long-term dynamics of both antigenic and non-antigenic properties of influenza virus.

## 1. Introduction

Influenza vaccines are reviewed annually to make strain recommendations for future vaccines [1]. This is necessary because the influenza virus constantly accumulates mutations in its haemagglutinin (HA) protein to escape antibodies (Abs) elicited against the virus through both natural infections and vaccinations. Antibodies recognize specific regions (epitopes) on the HA protein. However, amino acid changes occur frequently in these regions by mutation [2,3]. New variants (or strains) of influenza viruses with different antigenicities result from such amino acid changes.

As a result of these mutations, influenza A and B viruses cause seasonal epidemics that are responsible for serious illnesses in 3–5 million people, resulting in about 250 000 to 500 000 deaths worldwide [1,4,5]. In 1952, the World Health Organization (WHO) established the Global Influenza Surveillance Network, with 112 national influenza centres in 82 countries, to monitor the emergence and spread of influenza virus strains [6]. The network typically characterizes antigenic properties of these strains by using the haemagglutination inhibition (HI) assay. Primarily, researchers perform HI assays of circulating strains using reference sera generated in organisms such as ferrets, and then analyse the resulting data to assist in recommending strains (of influenza A and B viruses) for incorporation into vaccines for subsequent influenza seasons [1,7–9].

The HI assays rely on the ability of the antibodies found in sera obtained from animals infected with influenza virus to prevent binding of virus to red cells (agglutination). HI assay results (titres) form the bases for comparing and characterizing strains, ascertaining antigenic similarity between strains and making informed decisions on future vaccine strains. However, they are not always as informative as desired. Indeed, derivative measures of antigenic similarity between strains such as the normalized HI titre (NHT) and the Archetti–Horsfall measure are not always good indicators of the relatedness of pairs of strains [10–14]. For instance, the use of NHT sometimes fails to capture an accurate antigenic relatedness between strains, leading to incorrectly identifying up to 95% of strains as antigenically similar [15,16].

Recently, a biophysical model of the HI titres developed by Ndifon [9] revealed that in addition to antigenic factors (e.g. the affinity of antibodies for virus), which are typically of greatest interest in influenza surveillance, non-antigenic factors (e.g. concentration of red cells and of virus used in HI assays, and avidity of virus for red cells) also determine the titres. In effect, this limits the efficient use of the titres for measuring antigenic similarities among influenza strains. This suggests the need to systematically decouple antigenic and non-antigenic contributions to better harness HI titres for accurate antigenic comparisons between virus strains.

In this paper, we present a method using Bayesian statistics for a systematic decoupling of antigenic and non-antigenic factors contributing to HI titres. In addition to the probabilistic nature of the Bayesian method which allows it to account for randomness in experimental measurements, it also makes use of prior knowledge of the values of antigenic and non-antigenic variables governing HI assays. In this way, the Bayesian framework enables inference of empirically realistic values of the antigenic and non-antigenic variables from HI titres. In order to apply the methods, antigenic data on influenza A (H3N2) viruses which have been experimentally generated between 1968 and 2016 were compiled. The method is then applied to these data to estimate non-antigenic variables as well as concentrations and affinities of Abs derived from animals infected with the influenza A (H3N2) virus. The results of this application of the method reveal its potential to complement other analytical methods used in global influenza surveillance.

## 2. Results

### 2.1. Bayesian method for inferring antigenic and non-antigenic variables from haemagglutination inhibition titres

Let *H*^*XY*^ denote a measured HI titre of strain *X* relative to strain *Y*, *A*^*Y*^ denote the concentration of antibodies obtained using strain *Y*, *K*^*XY*^ denote the average affinity of those antibodies for antigens of strain *X* and *J*^*X*^ denote a dimensionless quantity that accounts for non-antigenic variables affecting the titres. Note that *J*^*X*^ is inversely correlated with the equilibrium association constant for virus–red cell bonds, which in turn is negatively correlated with the cleaving of HA by neuraminidase [9]. This implies that the addition of neuraminidase to the HI assay would affect the value of *J*^*X*^. For a collection of titres of *m* strains relative to sera raised against *n* strains, a Bayesian inference procedure is developed to deduce the concentration of antibodies, average affinities of those antibodies for virus antigens and the non-antigenic variables. The HI titre (*H*^*XY*^) is linearly related to the concentration of antibodies (*A*^*Y*^), the affinities of those antibodies for virus antigens, *K*^*XY*^, and other non-antigenic factors on a logarithmic scale [9]. Therefore, a logarithmic transformation of a collection of HI titres can be expressed as follows (see Methods):

2.1 $$\log(H^{XY})=\log(A^{Y})+\log(K^{XY})+\log(J^{X}),$$

where *H*^*XY*^ is the collection of all HI titres of *m* strains relative to sera raised against *n* strains, *A*^*Y*^ is the corresponding concentrations of antibodies (Abs) in each of *n* sera, *K*^*XY*^ is the collection of average affinities of those antibodies and *J*^*X*^ denotes the non-antigenic variables. The base of the logarithms in equation (2.1) is taken as two to reflect the twofold dilutions of sera in HI assays. For convenient referencing, we denote the logarithmic transformations to the base two of the variables in equation (2.1) simply by the respective variables, namely *H*^*XY*^, *A*^*Y*^, *K*^*XY*^ and *J*^*X*^.

The current collection of HI titres is normally distributed (electronic supplementary material). Therefore, taking *H*^*XY*^ to be normally distributed with mean *A*^*Y*^ + *K*^*XY*^ + *J*^*X*^ and variance 1/*τ* (where *τ* is a precision parameter), the likelihood of *H*^*XY*^ is given by

2.2 $$P(H^{XY}|A^{Y},K^{XY},J^{X},\tau )=\Pi_{X=1}^{m}\Pi_{Y=1}^{n}\frac{\sqrt{\tau }}{\sqrt{2}\pi }{e^{-\tau /2(H^{XY}-(A^{Y}+K^{XY}+J^{X}))}}^{2}.$$

By sampling distributions of means, we expect *A*^*Y*^ + *K*^*XY*^ + *J*^*X*^ to be normally distributed while the precision parameter, *τ,* is gamma distributed for large samples [17]. Consequently, it suffices to make the following definitions:

1. $A^{Y}∼N(\mu_{A},\text{1/}\tau_{A})$, where the hyperparameter *μ_A_* is the mean of *A*^*Y*^, and its prior density is also normally distributed with mean *μ′_A_* and variance 1/*τ′_A_*, both of which are known. The prior density of the hyperparameter *τ_A_* is gamma distributed with known shape *α_A_* and rate *β_A_*.
2. $K^{XY}∼N(\mu_{K},\text{1/}\tau_{K})$, where the parameter *μ_K_* is the mean of *K*^*XY*^, and its prior density is normally distributed with mean *μ′_K_* and variance 1/*τ'_K_*, both of which are known. The prior density of the parameter *τ_K_* is gamma distributed with known shape *α_K_* and rate *β_K_*.
3. $J^{X}∼N(\mu_{J},\text{1/}\tau_{J})$, where the parameter *μ_J_* is the mean of *J*^*X*^, and its prior density is normally distributed with mean *μ'_J_* and variance 1/*τ'_J_*, which are known. The prior density of the hyperparameter *τ_J_* is gamma distributed with known shape *α_J_* and rate *β_J_*.
4. *τ *∼ Γ(*α*, *β*), where both shape *α* and rate *β* are known variables which are expected to be affected by the precisions of the estimates of *A*^*Y*^, *K*^*XY*^ and *J*^*X*^. These are reflected in the derivation of the full conditional distributions of *τ* and the other precision parameters (*τ_A_, τ_K_* and *τ_J_*).

Using Bayes' Theorem, given an HI titre dataset, it can readily be shown that the full posterior density of all the parameters is given by

2.3 f(θ|H)αf(H|θ)f(θ),

where $\theta =(A^{Y},K^{XY},J^{X},\mu_{A},\mu_{K},\mu_{J},\tau )$ and *H* is the collection of all the HI titres. Note that *f*(*H*|*θ*) is the likelihood and *f*(*θ*) is the prior.

Therefore, it can readily be shown that the full conditional distributions of the concentrations of antibodies, the average affinities of those antibodies for viruses and the non-antigenic parameter are given by

1. $f(A^{Y}|\theta_{A},\lambda_{A})∼N(\theta_{A},1/\lambda_{A})$,
2. where $\theta_{A}=(S_{1}+\mu_{A}\tau_{A})/(m\tau +\tau_{A})$, $\lambda_{A}=m\tau +\tau_{A}$ and $S_{1}=\tau \sum_{X=1}^{m}\left(H^{XY}-K^{XY}-J^{X}\right)$,
3. $f(K^{\mathrm{XY}}|\theta_{K},\lambda_{K})∼N\left(\theta_{K},\frac{\text{1}}{\lambda_{K}}\right)$,
4. where $\theta_{K}=(S_{2}+\mu_{K}\tau_{K})/(\tau +\tau_{K})$, $\lambda_{K}=\tau +\tau_{K}$ and $S_{2}=\tau (H^{XY}-A^{Y}-J^{X})$
5. $f(J^{X}|\theta_{J},\lambda_{J})∼N\left(\theta_{J},\frac{\text{1}}{\lambda_{J}}\right)$,
6. where $\theta_{J}=(S_{3}+\mu_{J}\tau_{J})/(n\tau +\tau_{J})$, $\theta_{J}=n\tau +\tau_{J}$ and $S_{3}=\tau \sum_{Y=1}^{n}\left(H^{XY}-A^{Y}-K^{XY}\right)$.

Details of the derivations of the above full conditional distributions along with the distributions of the other parameters of the model are presented in the electronic supplementary material. Thus, with these established distributions, it is possible to employ the Gibbs sampler [18,19] for accurate inference of the HI titre parameters.

### 2.2. Model evaluation for estimating haemagglutination inhibition titres

In order to apply the method, 3969 HI titres corresponding to 3969 influenza A (H3N2) strain–serum pairs were obtained from published datasets [20–26]. The sera were raised using 117 distinct strains isolated between 1968 and 2015. From these datasets, we inferred the distributions of strain- and serum-specific parameters that underlie each HI titre. To ascertain the accuracy of the model, the inferred distributions for each combination of strain- and serum-specific parameters were used to estimate the corresponding HI titre. Comparing the estimated versus the actual titres on the logarithmic scale, we obtained very small values of mean absolute percentage error (MAPE) of 7.41 × 10^−04^ and mean absolute error (MAD) of 5.53 × 10^−03^, and a perfect correlation (*r *= 1, *p < *2.2 × 10^−16^). In addition, a Mann–Whitney test showed that the estimated and actual values of the HI titres are statistically indistinguishable (*p *= 0.91).

The Bayesian method estimated the concentrations of Abs in the 117 sera under consideration. These estimates range from 0.11 nM to 0.13 µM, and have a median of about 59.84 nM (electronic supplementary material, table S1) consistent with reported empirical values of Ab concentrations [12]. Similarly, the Bayesian method yielded estimates of the affinities of antibodies found in the considered sera that are consistent with values reported in the literature [27]. Specifically, the estimated affinities ranged between 20.32*k* per M to 27.42*G* per M, with a median of about 2.96*M* per M (electronic supplementary material, table S2). These values are in line with affinities of monoclonal antibodies elicited against the influenza A (H3N2) subtypes as measured in previous studies [9,28]. Affinities were significantly higher for homologous virus–antibody interactions than for heterologous virus–antibody interactions (median affinity approx. 13.36*M* per M versus 2.84*M* per M, *p* = 7.27 × 10^−12^). This indicates that antibodies generated against a particular strain have higher affinities for the same strain compared to other strains, which is in line with empirical expectations.

Furthermore, we checked whether there is asymmetry in the relationship between pairs of strains with respect to the affinities of their induced antibodies. For each pair of strains, we compared the affinity of antibodies generated against one strain for the other strain versus the affinity of antibodies generated against the latter strain for the former strain. A Mann–Whitney test showed that there is no statistically significant difference between both sets of affinities (*p *= 1). For instance, if Abs are raised against strain *V*1, then their affinity for an antigenically distinct strain, *V*2, is comparable to the affinity of Abs raised against *V*2 for *V*1. This suggests that antibody affinity does not in general induce asymmetry in the antigenic relationships between pairs of influenza A (H3N2) virus strains.

### 2.3. Model evaluation for distinguishing the sources of red blood cells in haemagglutination inhibition assays

**Figure 1.**
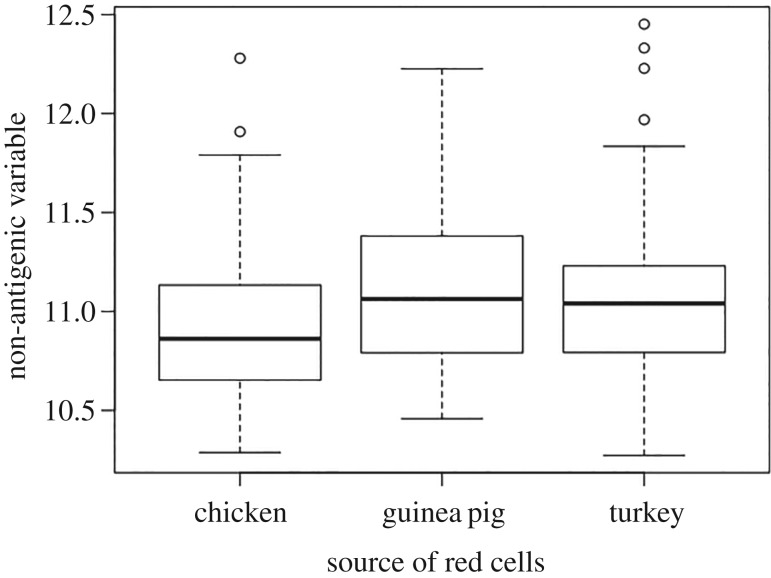
Variability of values of the non-antigenic variable across sources of red cells used in HI assays. Plotted values are log-transformed averages of Bayesian inference estimates.

In decomposing HI titres, both antigenic and non-antigenic variables are well separated. The Bayesian method estimated non-antigenic variables for viruses isolated between 1968 and 2016. The estimated values varied between 1237.27 and 5600.13, with a median of 1999.17 (electronic supplementary material, table S3). The collection of HI titres from different studies [20–26] were obtained using different organismal sources of red cells in HI assays, namely chicken, turkey and guinea pig. The estimated values of the non-antigenic variables varied in some instances with the source of red cells (figure 1). In particular, the estimated values corresponding to chicken red cells were significantly lower than those for turkey red cells (*p *= 2.24 × 10^−03^) and those for guinea pig red cells (*p *= 8.97 × 10^−03^). Of note, the estimated values for chicken red cells were the least variable, suggesting that use of these red cells might introduce less confounding, non-antigenic variation into the analysis of HI titres. The differences observed among the values of non-antigenic variables obtained using different species of red cells make sense. The non-antigenic variables are inversely correlated with virus avidity for red cells [9], and these cells may differ in their virus receptor binding sites dependent on their organismal source. These results contrast with those of Smith and co-workers [20], who found no differences between HI titres obtained using different species of red cells. This discrepancy might derive from the fact that here we focus our comparisons on the non-antigenic variables, which are expected to be better correlated with the species of red cells.

### 2.4. Bayesian inference reveals oscillatory dynamics in non-antigenic variables during influenza virus evolution

**Figure 2.**
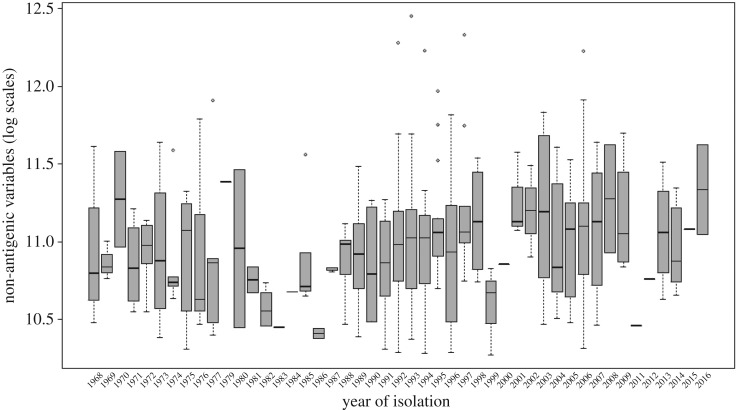
Apparent oscillation of values of the non-antigenic variable during 48 years of virus evolution. Plotted values are log-transformed averages of Bayesian inference estimates.

**Figure 3:**
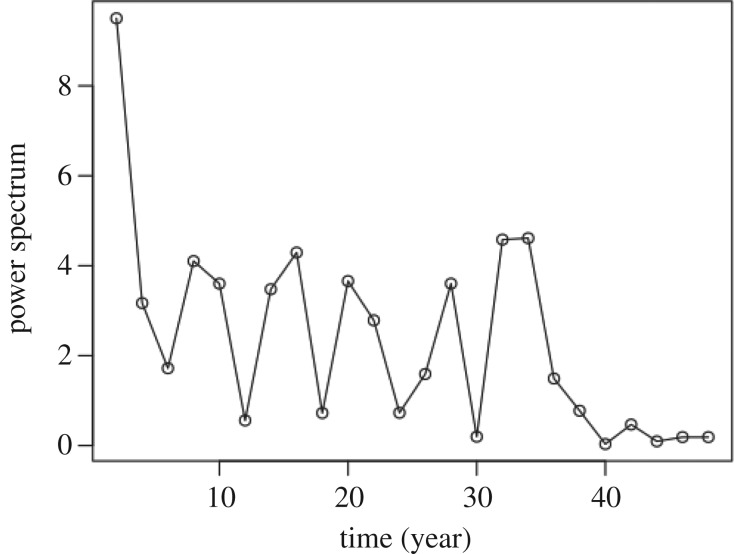
Power spectrum of median values of the non-antigenic variable estimated from HI titres collected during 48 years of influenza surveillance.

Our analysis makes it possible to study the long-term dynamics of non-antigenic variables associated with the evolution of influenza viruses. Performing this analysis, we find that the values of the non-antigenic variable estimated over a 48-year period (from 1968 to 2016) are positively correlated with the year of virus isolation (*r* = 0.15, *p* = 0.003; figure 2). Because the non-antigenic variable is inversely correlated with virus avidity for red cells [9], this result implies that avidity has decreased over time during the considered period. Recent reports suggest influenza A/H3N2 virus has acquired avidity-decreasing changes to its haemagglutinin [29]. In addition, the estimated values of the non-antigenic variable appear to oscillate (figure 2). In a further analysis, we investigated the statistical significance of the oscillatory components of these dynamics. In particular, we performed a Fourier discrete decomposition of the medians of the estimated values of the non-antigenic variables and found several prominent peaks in the resulting power spectrum (figure 3). We used a permutation test to investigate the statistical significance of each of these peaks based on the null hypothesis that peaks occur randomly at each period. The test is performed in a way that preserves the periodic frequencies of the power spectrum.

To determine the significance of a particular peak, we find the number of the corresponding peaks in the power spectrum obtained from Fourier decomposition of permuted samples that are greater than or equal to the focal peak, and then we divide this number by the total number of permuted samples considered. For convenience and accuracy, 500 permuted samples were used. A similar approach was used in a previous study to analyse a short time series microarray data [30]. However, while we compare power spectra here, that previous study compared periodograms, although the two are directly proportional to each other. Strikingly, only one peak, occurring at a period of 2 years, was found to be significant at a 5% level of significance.

Significance of the peak with a 2-year period suggests that non-antigenic factors such as the virus avidities for red cells are altered as part of the systematic changes influenza viruses undergo to evade neutralizing antibodies during infection. This provides further support for the hypothesis [31,32] that changes in virus receptor avidity partly account for differences among evolving influenza strains that enable their escape from antibodies.

## 3. Discussion

HI assays are widely used in global surveillance of the antigenic properties of influenza viruses, enabling the design of seasonal influenza vaccines. It was previously suggested that use of the assay's outputs (i.e. HI titres) for influenza surveillance could be further improved by accounting for the influence of non-antigenic factors on the titres [9,32]. In previous work [9], we developed a biophysical model linking HI titres to the underlying antigenic (i.e. concentrations of antibodies found in sera used in HI assays and the affinities of those antibodies for influenza viruses) and non-antigenic (i.e. dependent on the avidity of influenza viruses for red cells used in HI assays) variables. Here, building on our previous results, we developed a Bayesian model for inferring the values of those antigenic and non-antigenic variables from HI titres.

We analysed the values of the non-antigenic variables inferred by fitting the Bayesian model to HI titres collected over a 48-year time period. We find that these values contain one oscillatory component with a period of 2 years. The 2-year oscillation coincides approximately with the timescale on which new antigenic variants of influenza viruses emerge and replace older variants [33]. This suggests that virus avidity for red cells, which is an important determinant of the non-antigenic variable, might change on a similar timescale as the antigenic replacement of influenza virus strains. This is in line with the intriguing hypothesis [31,32] that changes in virus receptor avidities partly account for differences among evolving influenza strains which enable them to escape antibodies.

Antibody affinities estimated using the Bayesian model could provide a novel basis for improving the determination of antigenic differences between influenza viruses. This is particularly important because accurate determination of antigenic relatedness between circulating and vaccine strains is required to inform the design of effective vaccines. Previous estimates of antigenic relatedness have suffered from the lack of a proper account of the non-antigenic contributions to HI titres [16]. For instance, Li and co-workers [32] revealed differences produced in clustering influenza strains after accounting for non-antigenic variables. In particular, controlling for the avidity of viruses for red cells reassigned the influenza A strains A/FI/8/95, A/GE/A9509/95 and A/NL/18/94 into clusters different from those elucidated by Smith and co-workers without controlling for such confounding, non-antigenic variation [20,32]. Therefore, the ability to decouple non-antigenic contributions to HI titres using the described Bayesian model could benefit the application of HI titres for influenza surveillance. In particular, this has the potential to improve the ability to predict the antigenic future of influenza viruses, thereby aiding the design of more effective influenza vaccines.

Our analysis has several limitations. In particular, there were more model parameters than data points, which necessitated our use of Bayesian hyperparameters to constrain the inferred parameter values so that they occurred within physically realistic ranges. Therefore, both the realism of the inferred parameter values and the excellent fit of the model to data were expected. By contrast, our most striking result—i.e. the inferred values of the non-antigenic variable *J*^*X*^ oscillate with a 2-year period—was not predetermined but emerged from the data. This result suggests that, in addition to antigenic variables, the non-antigenic variable can also provide insight into influenza virus's antigenic evolution, in line with recent experimental and empirical observations [31,32].

## 4. Methods

### 4.1. Determination of haemagglutination inhibition titre

The experimental procedure used to determine the HI titre has been described previously [9]. Briefly, HI assays take advantage of the facts that influenza virus has the ability to agglutinate red blood cells and they lose this ability when incubated with sufficient amounts of neutralizing antibodies. In the assay, solutions containing a fixed amount of influenza virus and a fixed amount of red cells are mixed with serial twofold dilutions of antibody-containing serum. The serum is typically raised from experimentally infected animals (such as ferrets). After incubating these antibody–virus–red cell mixtures for a defined length of time, they are checked for the presence of agglutination. The HI titre is defined as the reciprocal of the highest serum dilution that inhibited agglutination. Typically, the HI titres are reported in a table with rows corresponding to viruses and columns corresponding to sera (electronic supplementary material, table S4). The entry found in row *X* and column *Y*, denoted *H*^*XY*^, is the highest dilution of serum *Y* that prevented agglutination of red cells by virus *X* in the HI assay. In this study, HI titres for influenza A (H3N2) viruses were taken from publicly available datasets collected between 1968 and 2016 [20–26]. They are reported in electronic supplementary material, table S4.

### 4.2. Bayesian inference of haemagglutination inhibition titre parameters

Given data, *D*, we define for a set of model parameters, *θ*, the joint distribution over all random quantities, *P*(*D*, *θ*) = *P*(*D*|*θ*)*P*(*θ*), where *P*(*θ*) is the prior distribution of *θ* and *P*(*D*|*θ*) is the likelihood. With known data *D*, Bayes' Theorem is applied to derive the posterior distribution:

4.1 $$P(\theta |D)=\frac{P(D|\theta )P(\theta )}{\int P(D|\theta )P(\theta )\;d\theta }.$$

Since the denominator of the above equation is integrating over the set of parameters, it suffices to derive an expression for the posterior distribution, *P*(*θ*|*D*), as follows:

4.2 P(θ|D)∝P(D|θ)P(θ).

We formulate the conditional distribution of form (4.2) from the set of measured HI titres. In particular, we find the full posterior distributions and make inference about the parameters of the distribution by employing Markov chain Monte Carlo methods [34]. For any given full conditional distribution, we proceed to construct Markov chain samples using the Gibbs sampler. Independent samples derived in this way are acceptable because they are produced from the entire domain of the posterior distribution (electronic supplementary material). We generated samples of sizes 100 000 each, discarding the first 2% of the sampled values as *burn-in* [35]. We observed no significant differences between parameter estimates obtained from independent samples of this size, justifying the choice of the sample size. We describe in detail the modelling and sampling of the HI titre parameters in the electronic supplementary material.

In order to assess the model fit, we performed a Mann–Whitney test (at 5% significance level) and also determined the MAPE as well as the MAD, thus establishing that there are no differences between the actual HI titres and the corresponding titres calculated based on the Bayesian inference procedure.

All statistical tests were performed at the 5% significance level. In addition, parameter values were compared with experimental values from the literature to confirm their realism.

## Supplementary Material

Supplementary Material

## Supplementary Material

Supplementary Table S1

## Supplementary Material

Supplementary Table S2

## Supplementary Material

Supplementary Table S3

## Supplementary Material

Supplementary Table S4

## Data accessibility

The datasets and supplementary materials supporting this article have been uploaded as part of the electronic supplementary material.
