# Supplementary material for: Bayesian inference of antigenic and non-antigenic variables from haemagglutination inhibition assays for influenza surveillance
Source: R Soc Open Sci. 2018 Jul 25;5(7):180113. doi: 10.1098/rsos.180113 (PMC6083687; doi:10.1098/rsos.180113)
Supplement: Supplementary Material [file rsos180113supp1.pdf]

Bayesian inference of antigenic and non-antigenic variables from haemagglutinin inhibition assays for influenza surveillance

Emmanuel S. Adabor and Wilfred Ndifon

Supplementary Material

Bayesian Inference of Parameters of Haemagglutinin Inhibition (HI) titre

Typically, the measured HI titre of a virus *X* relative to virus *Y* in an assay, denoted by H^{XY}, is as follows [1]:

$$H^{XY} = A^{Y} K^{XY} J^{x} \qquad \text{S1}$$

where A^{Y} is the concentration of antibodies found in *Y*-derived serum, K^{XY} is the average affinity of those antibodies for virus *X*, and J^{X} is a dimensionless quantity that account for the non-antigenic factors that affected the HI titre, H^{XY}. This quantity basically depends on the avidity of virus for red blood cells, concentration of virus and red blood cells.

A natural way to decouple both antigenic and non-antigenic parameters of the HI titre is to take the logarithmic transformation of the titre. The result of such transformation is:

$$\log\left(H^{XY}\right) = \log\left(A^{Y}\right) + \log\left(K^{XY}\right) + \log\left(J^{x}\right). \qquad \text{S2}$$

Thus, in subsequent analysis, each of these variables referred to is a log-transformed variable. The log-transformed HI titres of the currently curated data are normally distributed by the shapiro test (p=0.07). Therefore taking that the H^{XY} is normally distributed with mean A^{Y} + K^{XY} + J^{X} and defining its variance as the fraction $\frac{1}{\tau}$ (where τ is a precision parameter), we had the likelihood of the entire titres as:

$$P\left(H^{XY} \mid A^{Y}, K^{XY}, J^{X}, \tau\right) = \Pi_{X=1}^{m} \Pi_{Y=1}^{n} \frac{\sqrt{\tau}}{\sqrt{2\pi}} e^{\frac{-\tau}{2}\left(H^{XY} - \left(A^{Y} + K^{XY} + J^{X}\right)\right)^2}, \quad \text{S3}$$

where m is the number of viruses, n is the number of sera produced in the HI assays, and H represents the collection of the entire HI titres. However, we expect the sampling distribution of the mean, $A^Y + K^{XY} + J^X$ to be normally distributed (for large sample size) while the quantity τ (precision) is gamma distributed [2].

More formally, we make the following definitions of density functions:

1. $A^Y \sim N\left(\mu_A, \frac{1}{\tau_A}\right)$, where the hyperparameter μ_A is the mean of A^Y and its prior density is also normally distributed with mean μ'_A and variance $1/\tau'_A$ which are known. The prior density of the hyper parameter τ_A is gamma distributed with shape α_A and rate β_A which are also known.

2. $K^{XY} \sim N\left(\mu_K, \frac{1}{\tau_K}\right)$, where the parameter μ_K is the mean of K^{XY} and its prior density is also normally distributed with mean μ'_K and variance $1/\tau'_K$ which are known. The prior density of the parameter τ_K is gamma distributed with the shape α_K and rate β_K which are also known.

3. $J^X \sim N\left(\mu_J, \frac{1}{\tau_J}\right)$, where the parameter μ_J is the mean of J^X and its prior density is also normally distributed with mean μ'_J and variance $1/\tau'_J$ which are known. The prior density of the hyper parameter τ_J is gamma distributed with the shape α_J and rate β_J .

4. $\tau \sim Gamma(\alpha, \beta)$, where both shape α and rate β are known variables which are expected to be affected by the precisions of the estimates of A^Y, K^{XY} and J^X. These are reflected in the derivation of the full conditional distribution of τ and the other precision parameters (τ_A, τ_K and τ_J).

Note that if random a variable X is normally distributed with mean μ and variance σ^2, then its probability distribution function is given by:

$$f(x) = \frac{1}{\sqrt{2\pi\sigma^2}} e^{-\frac{1}{2}\left(\frac{x-\mu}{\sigma}\right)^2} . \qquad \text{S4}$$

Also, if a random variable X follows a gamma distribution, then the probability distribution function of X is given by:

$$f(x) = \frac{\beta^{\alpha}}{\Gamma(\alpha)} x^{\alpha-1} e^{-\beta x}, x > 0, \alpha > 0, \beta > 0 , \qquad \text{S5}$$

where $x > 0$, the shape $\alpha > 0$ and the rate $\beta > 0$.

For an entire collection of HI titres, H, it follows from applying the Bayes' Theorem that the full posterior density of all the parameters can be expressed as:

$$f(\theta \mid H) \, \alpha \, f(H \mid \theta) f(\theta) \qquad \text{S6}$$

where $\theta = (A^{Y}, K^{XY}, J^{X}, \mu_A, \mu_K, \mu_J, \tau,)$ and H is the collection of the entire HI titres. Note that $f(H \mid \theta)$ is the likelihood and $f(\theta)$ is the prior.

Therefore, expanding the expression to the right hand side of S6 results in the following:

$$\begin{gathered} f(\theta \mid H) \, \alpha \, \Pi_{X=1}^{m} \Pi_{Y=1}^{n} \sqrt{\tau} e^{\frac{-\tau}{2}\left(H^{XY} - A^{Y} - K^{XY} - J^{X}\right)^2} \cdot \Pi_{Y=1}^{n} \sqrt{\tau_A} e^{-\frac{\tau_A}{2}\left(A^{Y} - \mu_A\right)^2} \cdot \Pi_{X=1}^{m} \Pi_{Y=1}^{n} \sqrt{\tau_K} e^{-\frac{\tau_K}{2}\left(K^{XY} - \mu_K\right)^2} \\ \Pi_{X=1}^{m} \sqrt{\tau_J} e^{-\frac{\tau_J}{2}\left(J^{X} - \mu_J\right)^2} \cdot \tau^{\alpha-1} e^{-\beta\tau} \cdot e^{-\frac{\tau_A}{2}\left(\mu_A - \mu_A\right)^2} \cdot e^{-\frac{\tau_K}{2}\left(\mu_K - \mu_K\right)^2} \cdot e^{-\frac{\tau_J}{2}\left(\mu_J - \mu_J\right)^2} \\ \tau_A^{\alpha_A - 1} e^{-\beta_A \tau_A} \cdot \tau_K^{\alpha_K - 1} e^{-\beta_K \tau_K} \cdot \tau_J^{\alpha_J - 1} e^{-\beta_J \tau_J} \end{gathered} \qquad \text{S7}$$

From S7, it can be readily shown that the full conditional distribution of all the parameters, θ, are summarized as follows:

1. $f\left(A^Y \mid \theta_A, \lambda_A\right) \sim N\left(\theta_A, \frac{1}{\lambda_A}\right)$, where $\theta_A = \frac{S_1 + \mu_A \tau_A}{m\tau + \tau_A}$, $\lambda_A = m\tau + \tau_A$ and $S_1 = \tau \sum \left(H^{XY} - K^{XY} - J^X\right)$.

2. $f\left(K^{XY} \mid \theta_K, \lambda_K\right) \sim N\left(\theta_K, \frac{1}{\lambda_K}\right)$, where $\theta_K = \frac{S_2 + \mu_K \tau_K}{\tau + \tau_K}$, $\lambda_K = \tau + \tau_K$ and $S_2 = \tau\left(H^{XY} - A^Y - J^X\right)$.

3. $f\left(J^X \mid \theta_J, \lambda_J\right) \sim N\left(\theta_J, \frac{1}{\lambda_J}\right)$, where $\theta_J = \frac{S_3 + \mu_J \tau_J}{n\tau + \tau_J}$, $\lambda_J = n\tau + \tau_J$ and $S_3 = \tau \sum \left(H^{XY} - A^Y - K^{XY}\right)$.

4. $f(\tau \mid s, r) = Gamma(s, r)$, where the shape, $s = \frac{1}{2}mn + \alpha$ and the rate, $r = \frac{1}{2}\sum\sum\left(H^{XY} - A^Y - K^{XY} - J^X\right)^2 + \beta$.

5. $f\left(\mu_A \mid \theta_{\mu_A}, \lambda_{\mu_A}\right) \sim N\left(\theta_{\mu_A}, \frac{1}{\lambda_{\mu_A}}\right)$, where $\theta_{\mu_A} = \frac{\tau_A \sum A^Y + \mu'_A \tau'_A}{n\tau_A + \tau'_A}$ and $\lambda_{\mu_A} = n\tau_A + \tau'_A$.

6. $f\left(\mu_K \mid \theta_{\mu_K}, \lambda_{\mu_K}\right) \sim N\left(\theta_{\mu_K}, \frac{1}{\lambda_{\mu_K}}\right)$, where $\theta_{\mu_K} = \frac{\tau_K \sum\sum K^{XY} + \mu'_K \tau'_K}{mn\tau_K + \tau'_K}$ and $\lambda_{\mu_K} = mn\tau_K + \tau'_K$.

7. $f\left(\mu_J \mid \theta_{\mu_J}, \lambda_{\mu_J}\right) \sim N\left(\theta_{\mu_J}, \frac{1}{\lambda_{\mu_J}}\right)$, where $\theta_{\mu_J} = \frac{\tau_J \sum J^X + \mu'_J \tau'_J}{m\tau_J + \tau'_J}$ and $\lambda_{\mu_J} = m\tau_J + \tau'_J$.

8. $f(\tau_A \mid r_A, s_A) \sim Gamma(s_A, r_A)$, where the shape, $s_A = \frac{1}{2}n + \alpha_A$ and the rate $r_A = \frac{1}{2}\sum(A^Y - \mu_A)^2 + \beta_A$.

9. $f(\tau_K \mid r_K, s_K) \sim Gamma(s_K, r_K)$, where the shape, $s_K = \frac{1}{2}mn + \alpha_K$ and the rate, $r_K = \frac{1}{2}\sum\sum(K^{XY} - \mu_K)^2 + \beta_K$.

10. $f(\tau_J \mid r_J, s_J) \sim Gamma(s_J, r_J)$, where the shape, $s_J = \frac{1}{2}m + \alpha_J$ and the rate, $r_J = \frac{1}{2}\sum(J^X - \mu_J)^2 + \beta_J$.

The superscripts and subscripts of variables denote value of a variable for a particular virus. For instance, A^Y denotes the concentration of antibodies derived against virus *Y*. Additional details of Bayesian inferences are discussed in the literature [3,4].

Sampling the values of Parameters

Since the full conditionals have been determined, we proceed to construct Markov chain samples with the Gipps sampler [5,6]. We expect the samples to be acceptable since they are produced from the entire domain of the posterior distribution [7]. In addition, autocorrelation function plots of the samples of each parameter further supported the independence of the samples of estimates (Figures S1-S3). Given independent samples, large samples of size 100,000 were selected with 2% as *burn-in* [8]. Moreover, no significant differences between estimates of different simulations with the

chosen sample size were observed which motivates the choice of the sample size. We obtained the mean of the samples as the estimate of the HI titre parameters.

In order to guide the sampling within acceptable range of the HI titres, initial values for the priors were searched from the literature. In particular, Ndifon and coworkers [9] estimated that high concentrations of antibodies in antisera from influenza virus-infected animals are of the order of 1.67*n*M. Furthermore, the average affinities of such antibodies were also found to range from 6*M* per M to 1*G* per M for influenza A (H3N2) subtypes [1]. On the other hand, initial values of non antigenic variables were estimated from the viral avidities (up to 50ug/mL RDE) as reported in [10]. This is because the avidity of virus for red cells mainly composes the non-antigenic variables [10]. More specifically, utilizing the prior knowledge about the HI titre parameters in the literature, initial values of the parameters within the expected ranges were randomly generated from the log-normal distribution such that the means of the samples were similar to the estimates reported in the earlier studies. This log-normal distribution was selected since log-transforming such samples will result in a normal distribution required for the sampling and the inference as indicated in Equation S2. Furthermore, the estimates obtained in this manner compared favourably well with the literature when the HI titre data were compared with the titre from the aggregation of the estimates obtained by our methods described herein. In particular, the Mann-Whitney test (with 0.05 level of significance) showed that there were no statistically significant differences between the two titres (p = 0.91). R codes were written to perform all simulations.

Supplementary Figures

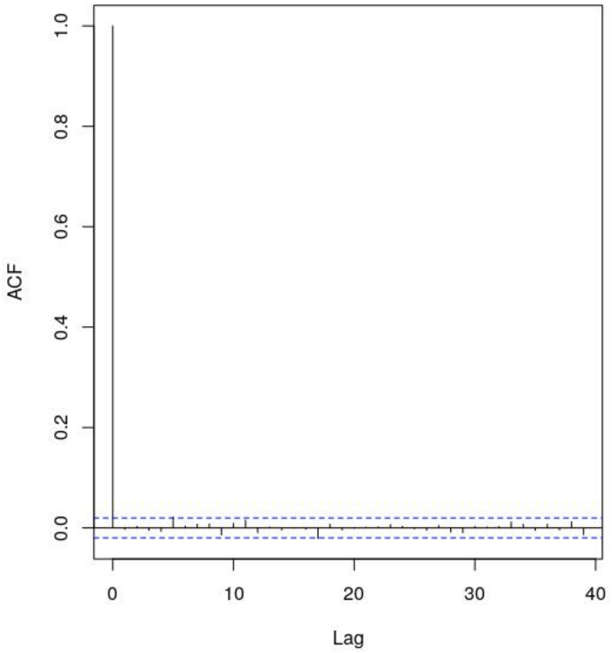

Figure S1: A sample of autocorrelation function (ACF) of Markov chain samples of concentration of an antibody.

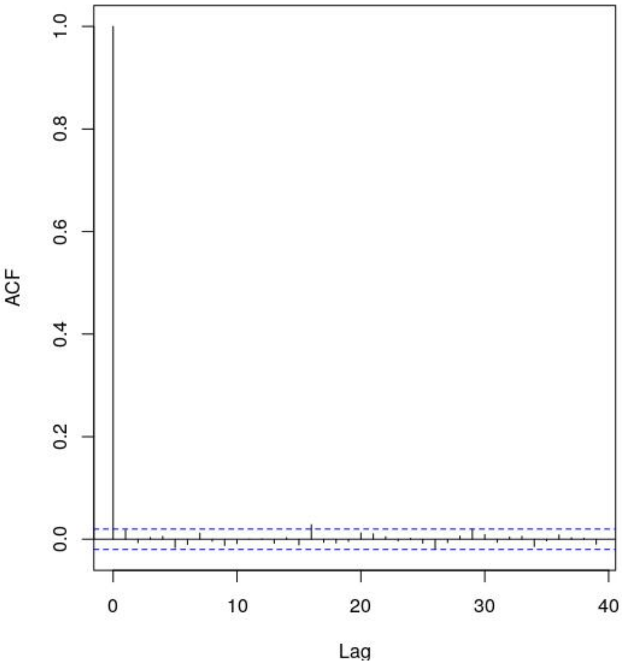

Figure S2: A sample autocorrelation function (ACF) of Markov chain samples of Affinities of an antibody for influenza A (H3N2) virus.

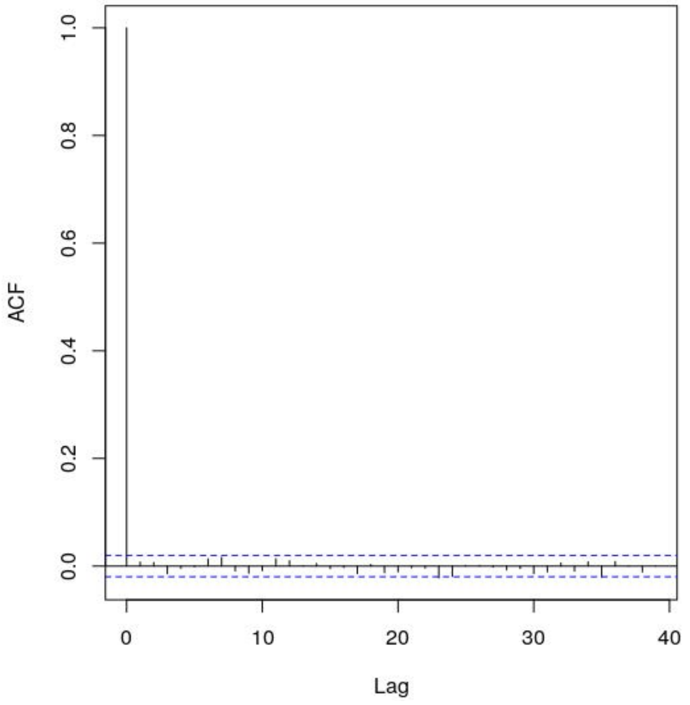

Figure S3: A sample autocorrelation function (ACF) of Markov chain samples of the non-antigenic variables associated an influenza A (H3N2) virus.

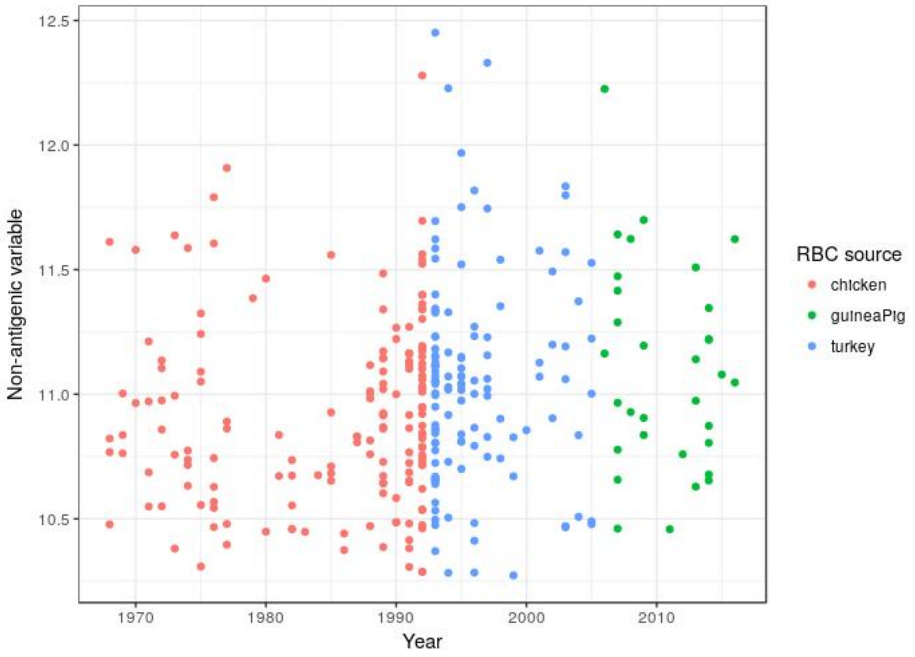

Figure S4. Sources of red blood cells in HI titres are distinguished by non-antigenic variables and Years. Non-antigenic variables are log-transformed averages of Bayesian inference estimates.

References

1. Ndifon W. 2011 New methods for analyzing serological data with applications to influenza surveillance. Influenza and Other Respiratory Viruses 5(3), 206-212. (doi:10.1111/j.1750-2659.2010.00192.x)

2. Dowdy S, Weardon S, Chilko D. 2004 *Statistics for research* (*3rd edition*). John Wiley & Sons, Inc., Hoboken, New Jersey.

3. Congdon P. 2003 Applied Bayesian Modelling. John Wiley & Sons Ltd. England.

4. Gregory PC. 2005 Bayesian Logical Data Analysis for the Physical Sciences. Cambridge University Press, UK.

5. Casella G, George EI. 1992 Explaining the Gibbs Sampler. The American Statistician 46(3), 167-174.

6. Robert CP, Casella G. 1999 Monte Carlo Statistical Methods. Springer-Verlag, New York.

7. Gilks WR, Richardson S, Spiegelhalter DJ. 1995 Markov Chain Monte Carlo in Practice. Chapman and Hall/CRC, London.

8. Geyer CJ. 1992 Practical Markov chain Monte Carlo. Statistical Science 7, 473-511

9. Ndifon W, Wingreen NS, Levin SA. 2009 Differential neutralization efficiency of haemagglutinin epitopes, antibody interference, and the design of influenza vaccines. Proc Natl Acad Sci USA 106(21), 8701-8706. (doi:10.1073/pnas.0903427106)

10. Li Y, Bostick DL, Sullivan CB, Myers JL, Griesemer SB, Stgeorge K, Plotkin JB, Hensley SE. 2013 Single Haemagglutinin Mutations that Alter both Antigenicity and Receptor Binding Avidity Influence Influenza Virus Antigenic Clustering. J. Virol 87(17), 9904-9910. (doi:10.1128/JVI.01023-13)
